# Supplementary figures and images for: Interaction between GRP78 and IGFBP-3 Affects Tumourigenesis and Prognosis in Breast Cancer Patients
Source: Cancers (Basel). 2020 Dec 18;12(12):3821. doi: 10.3390/cancers12123821 (PMC7767108; doi:10.3390/cancers12123821)

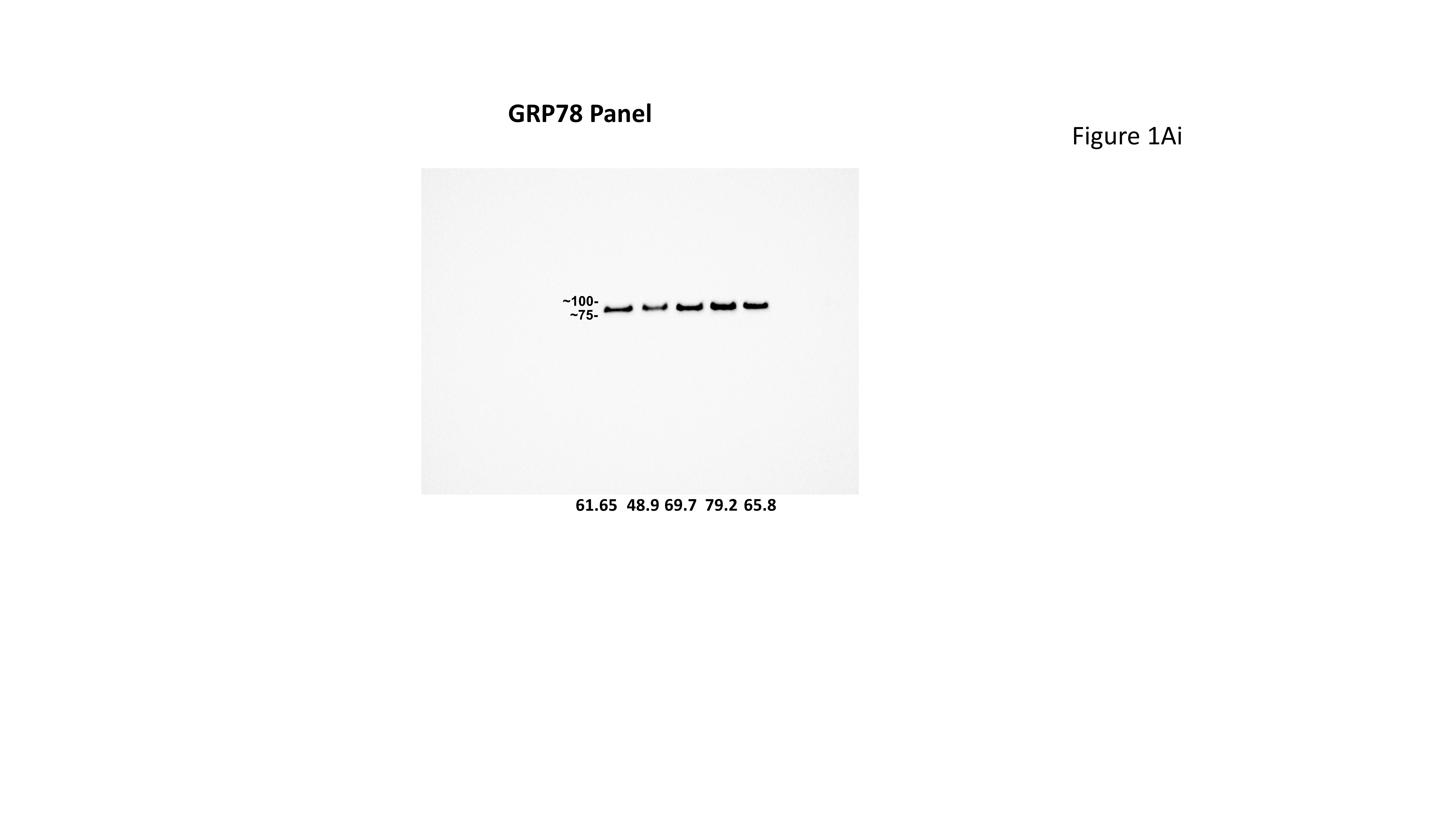

Supplement: Supplementary file 1 [file cancers-12-03821-s001.zip › original WB 20-10-30/Slide1.TIF]

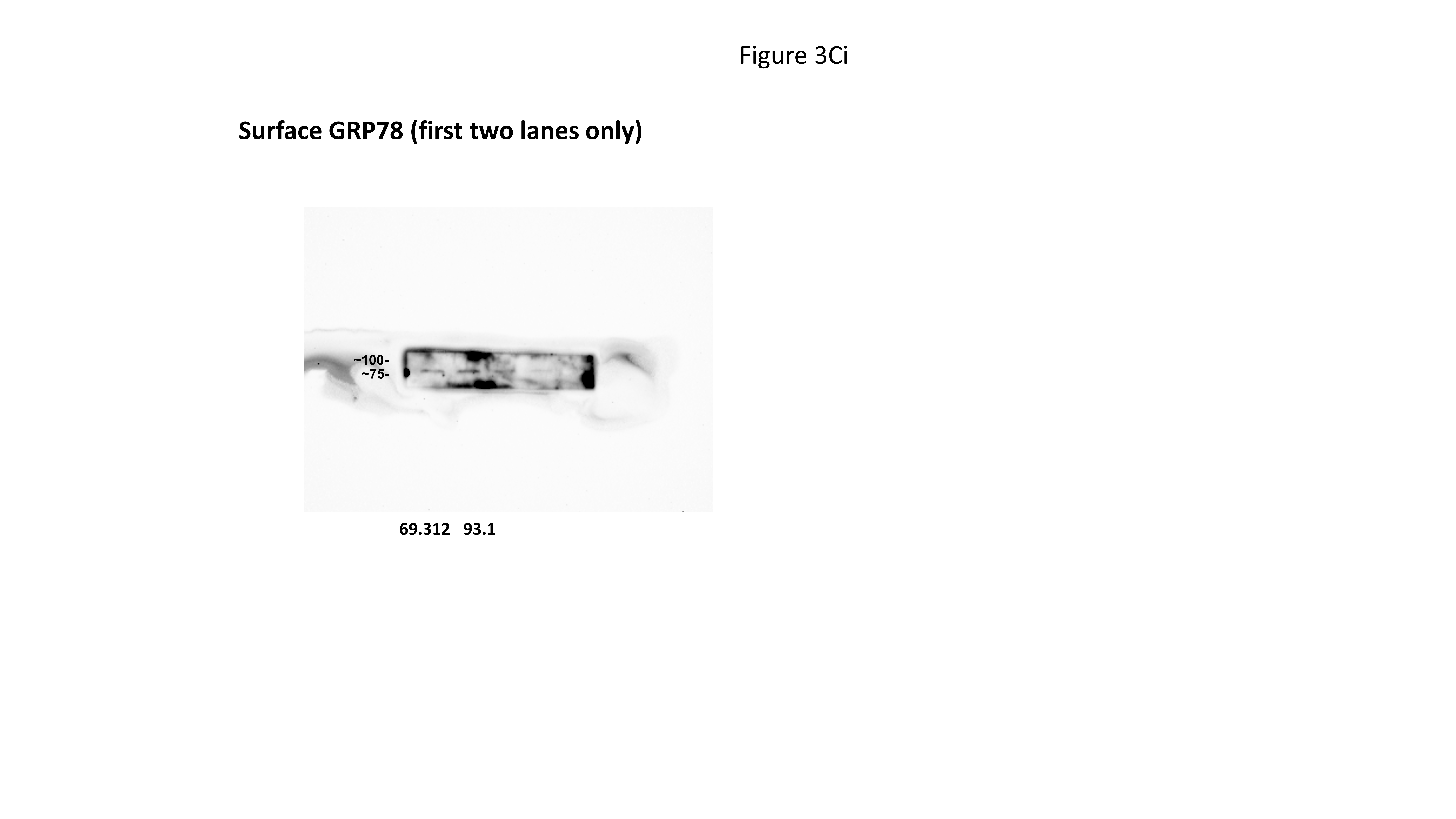

Supplement: Supplementary file 1 [file cancers-12-03821-s001.zip › original WB 20-10-30/Slide10.TIF]

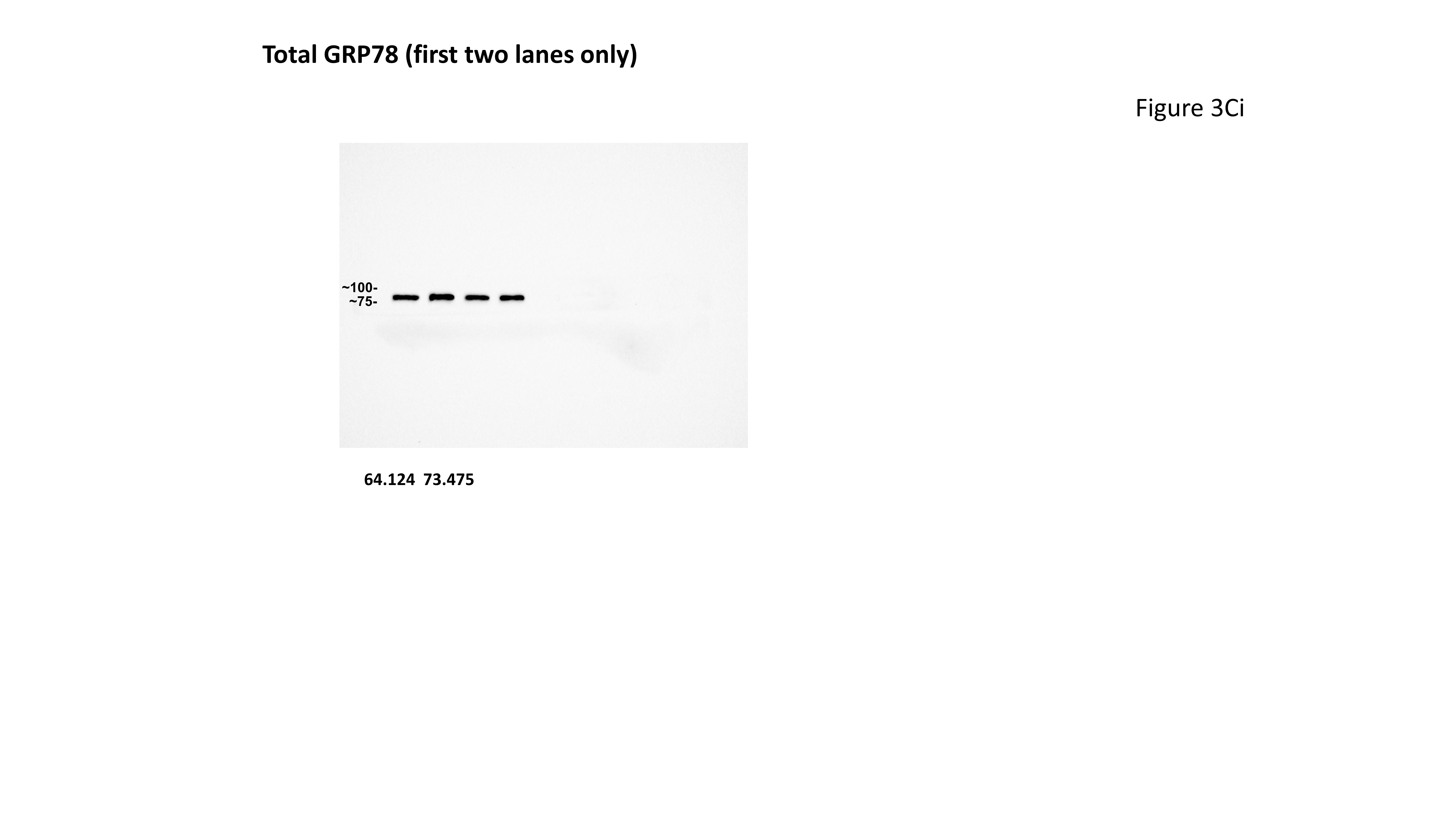

Supplement: Supplementary file 1 [file cancers-12-03821-s001.zip › original WB 20-10-30/Slide11.TIF]

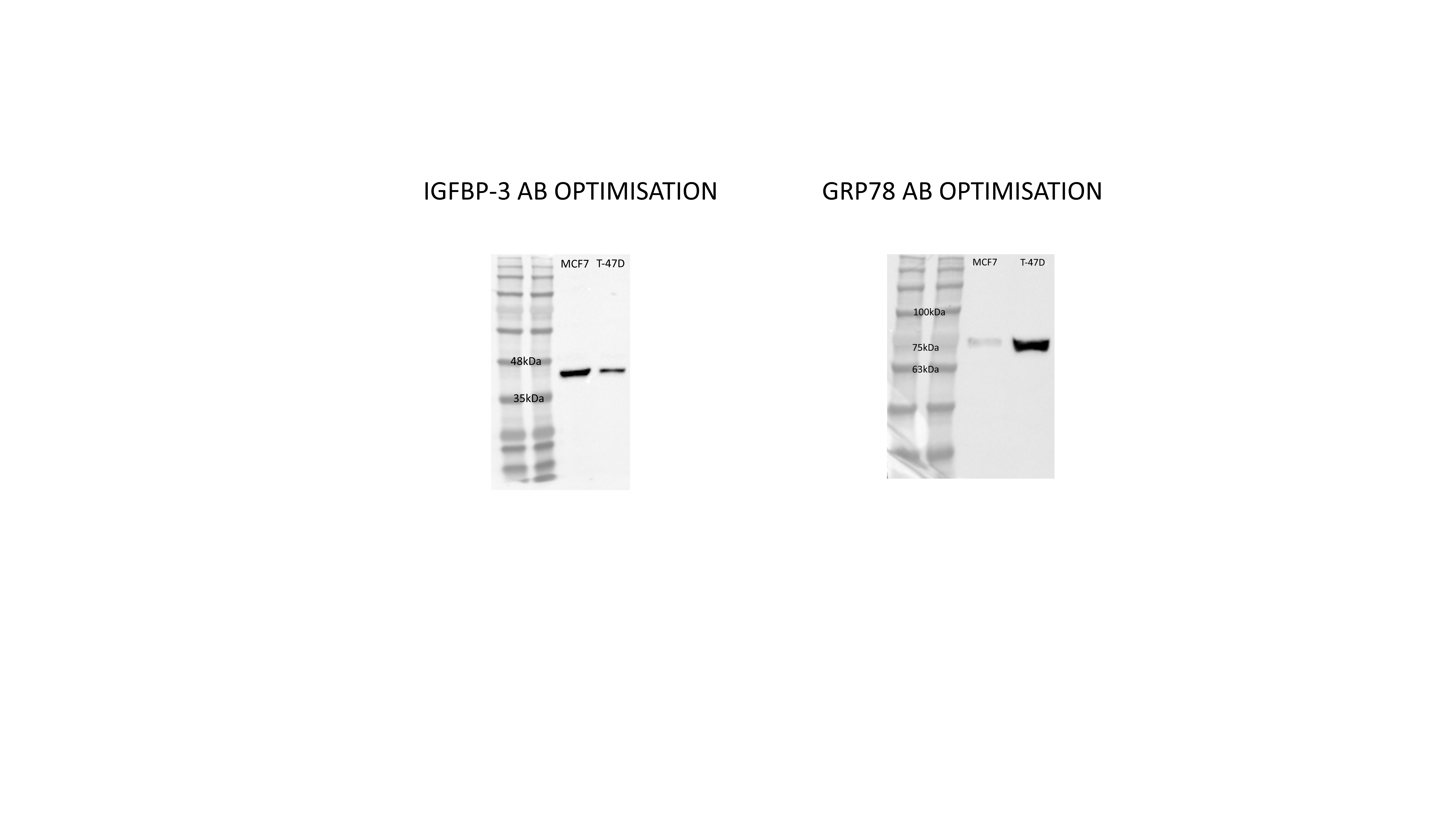

Supplement: Supplementary file 1 [file cancers-12-03821-s001.zip › original WB 20-10-30/Slide12.TIF]

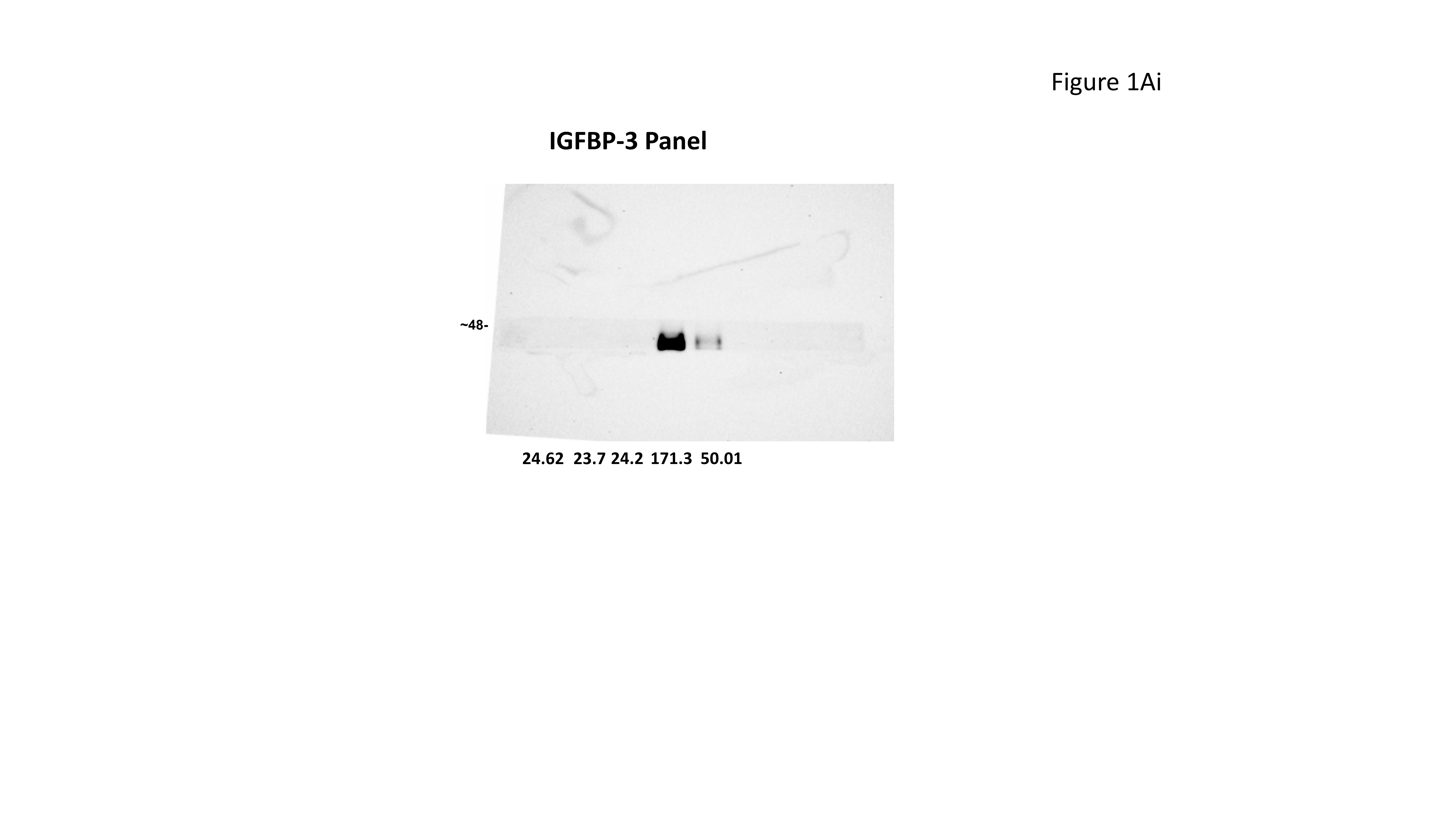

Supplement: Supplementary file 1 [file cancers-12-03821-s001.zip › original WB 20-10-30/Slide2.TIF]

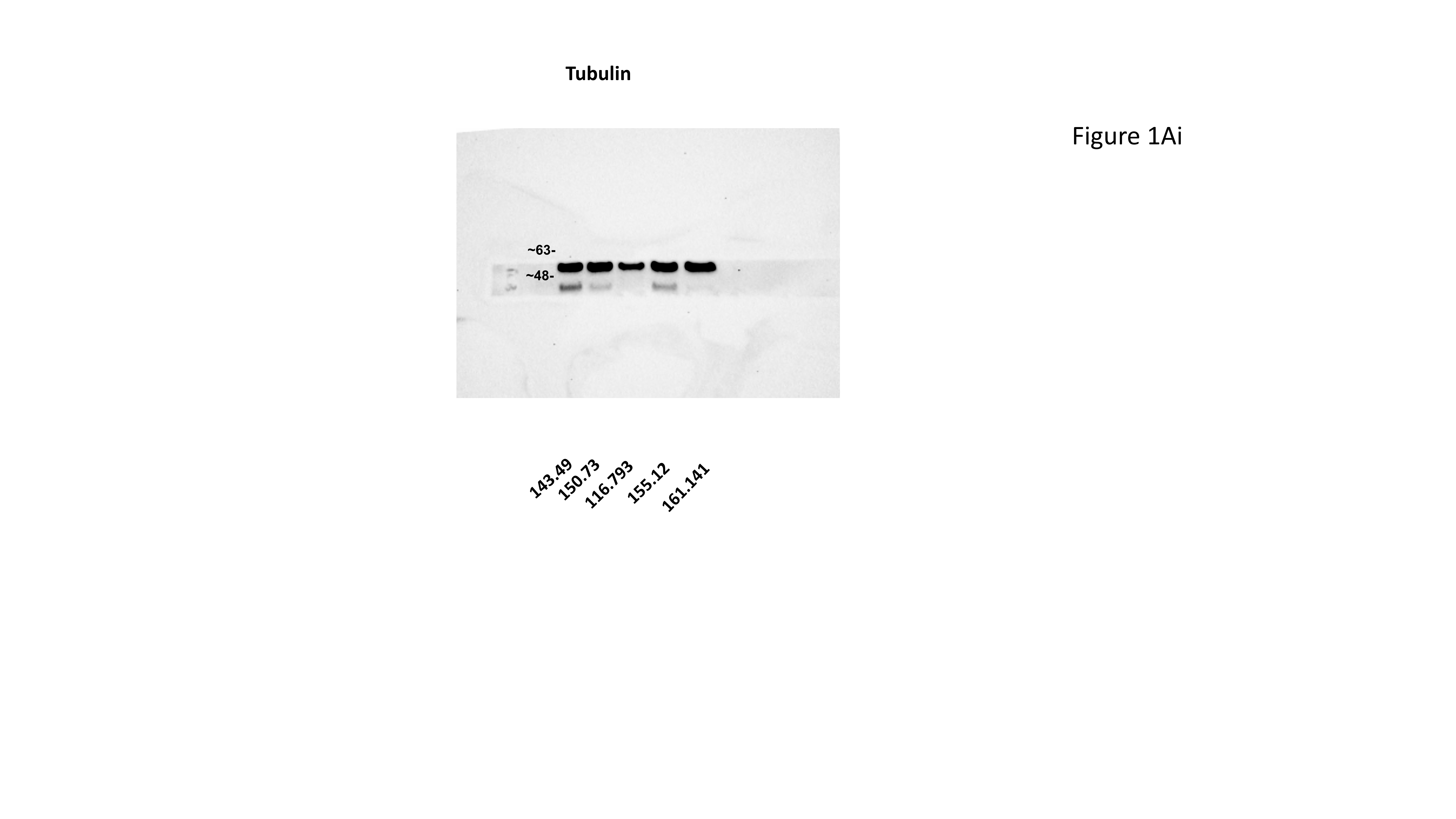

Supplement: Supplementary file 1 [file cancers-12-03821-s001.zip › original WB 20-10-30/Slide3.TIF]

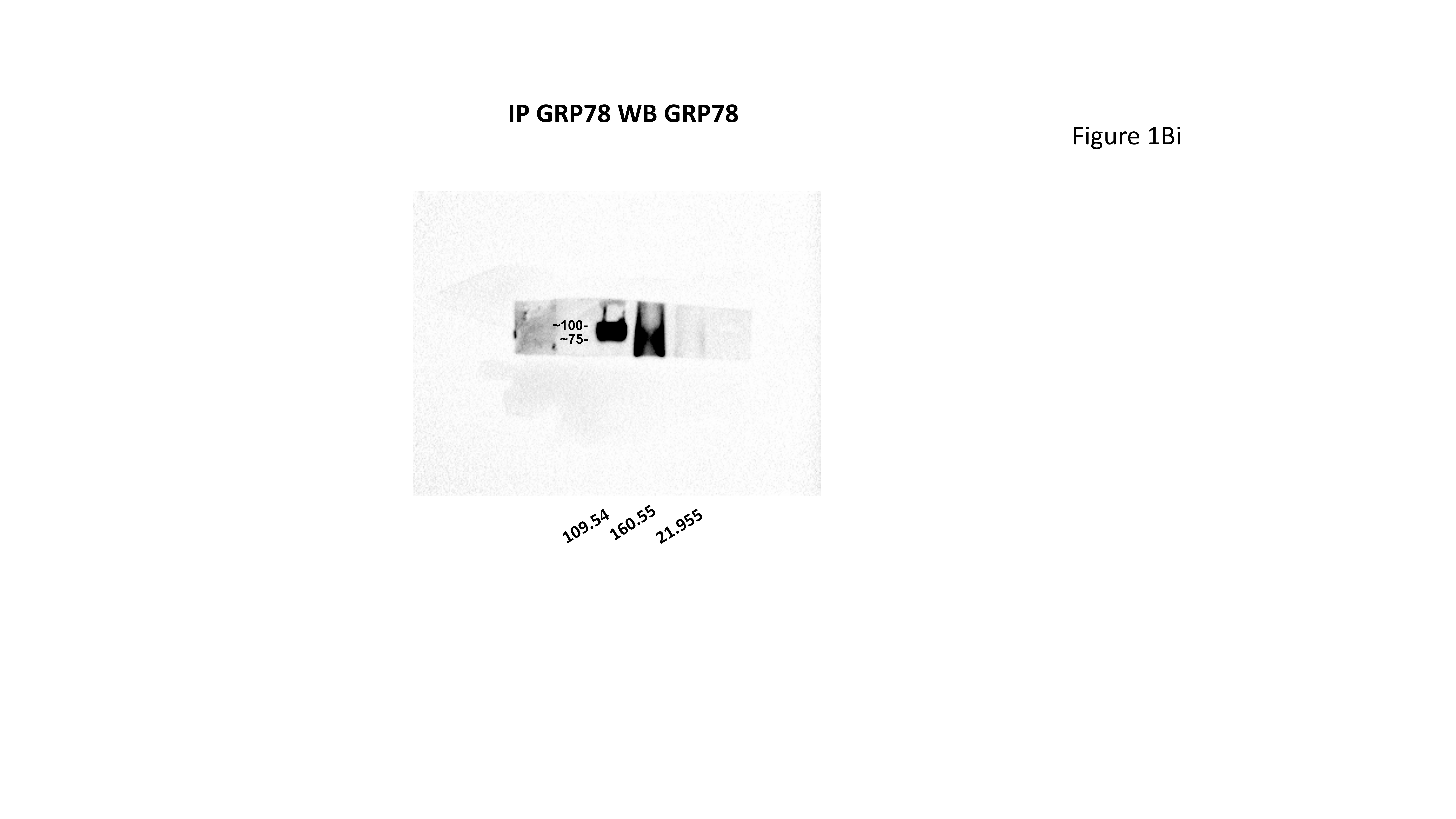

Supplement: Supplementary file 1 [file cancers-12-03821-s001.zip › original WB 20-10-30/Slide4.TIF]

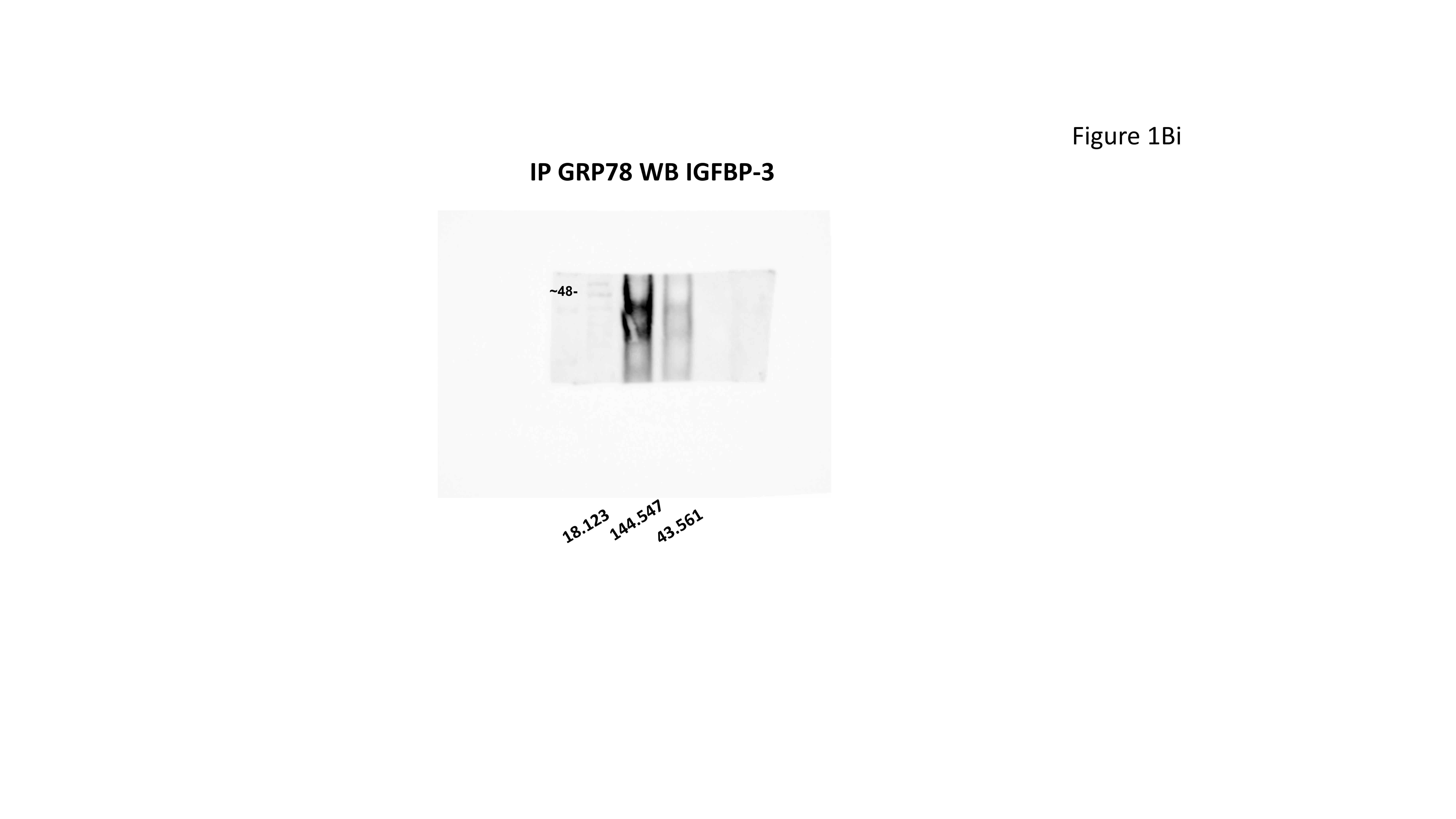

Supplement: Supplementary file 1 [file cancers-12-03821-s001.zip › original WB 20-10-30/Slide5.TIF]

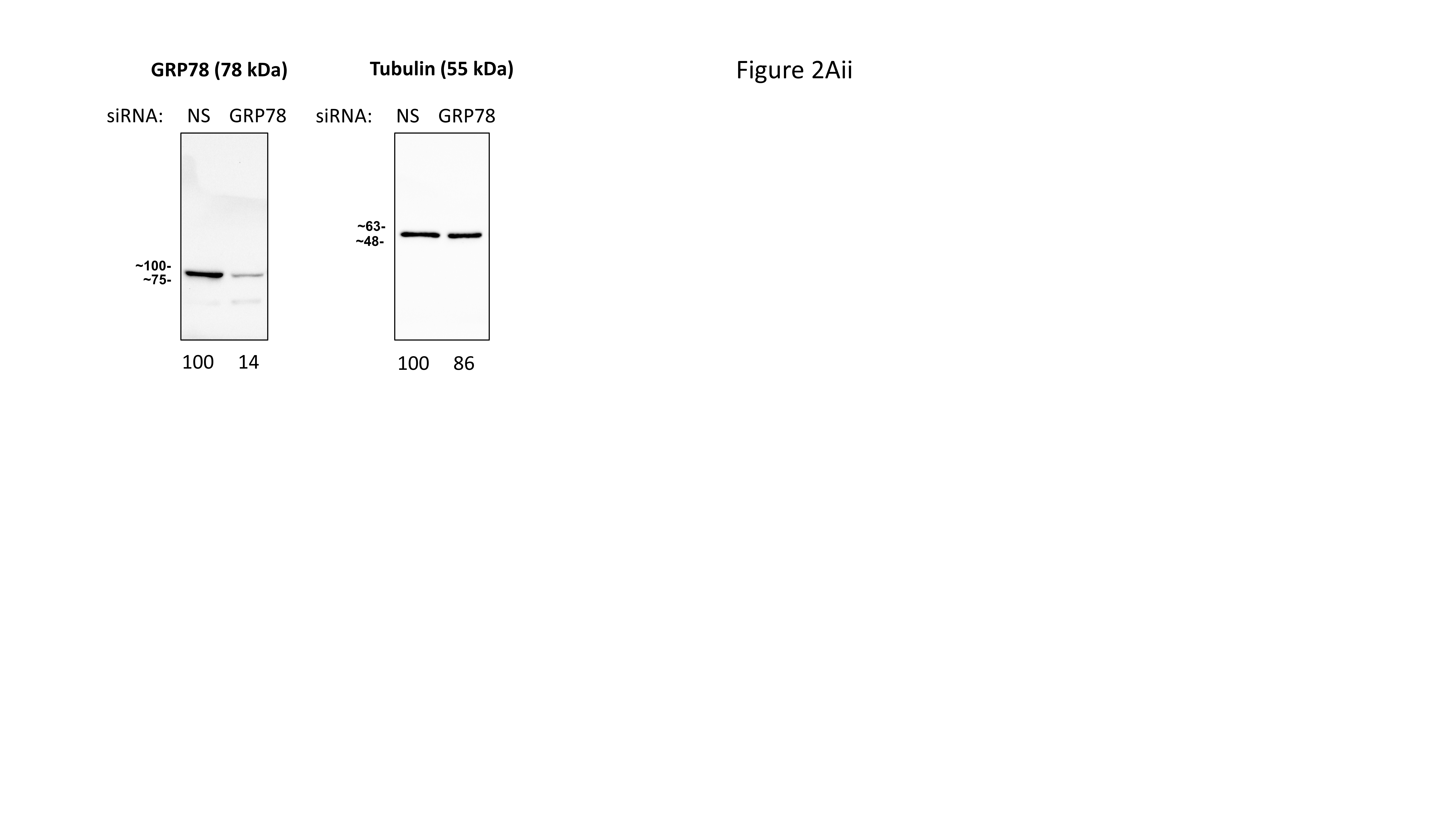

Supplement: Supplementary file 1 [file cancers-12-03821-s001.zip › original WB 20-10-30/Slide6.TIF]

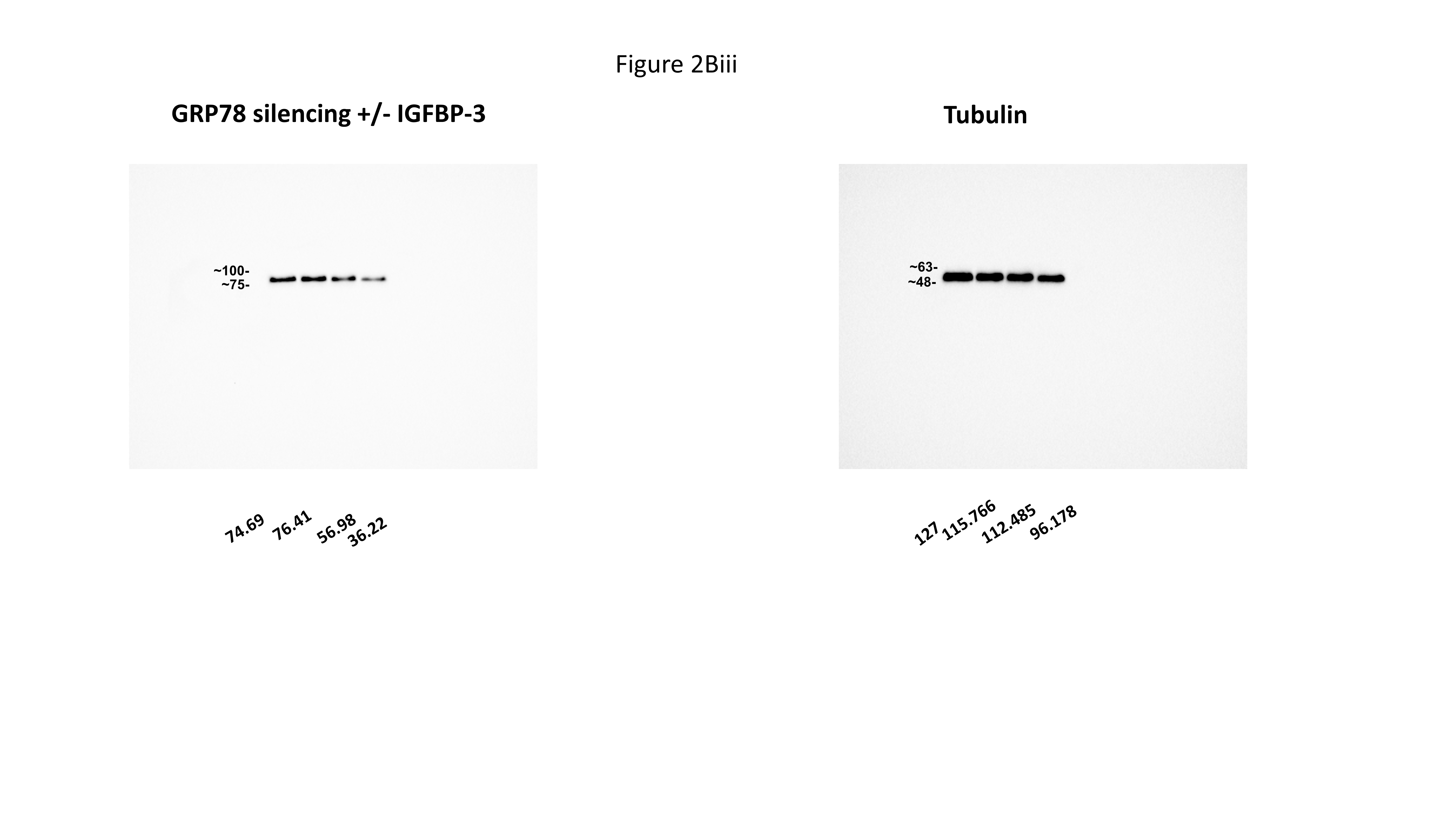

Supplement: Supplementary file 1 [file cancers-12-03821-s001.zip › original WB 20-10-30/Slide7.TIF]

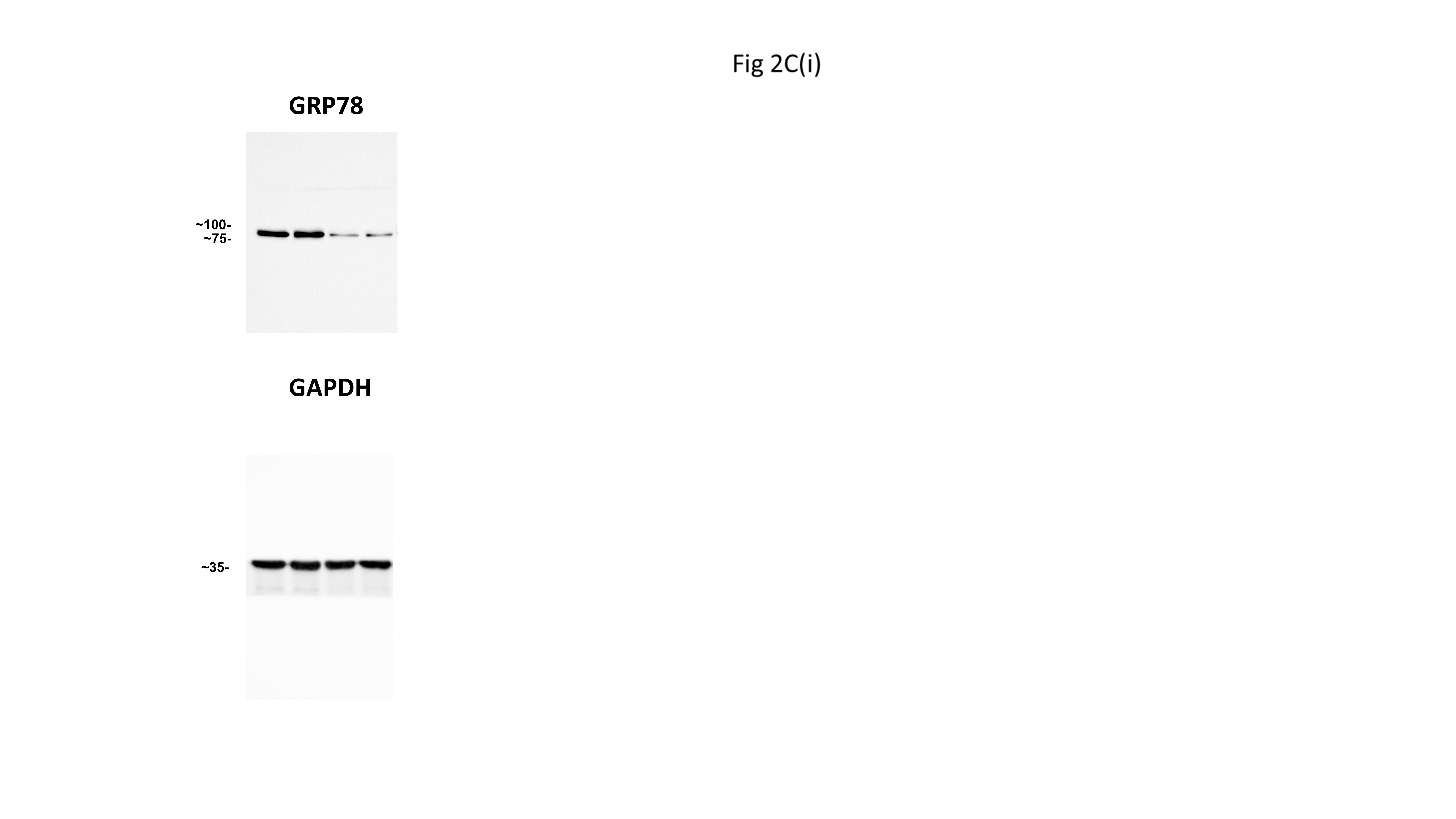

Supplement: Supplementary file 1 [file cancers-12-03821-s001.zip › original WB 20-10-30/Slide8.TIF]

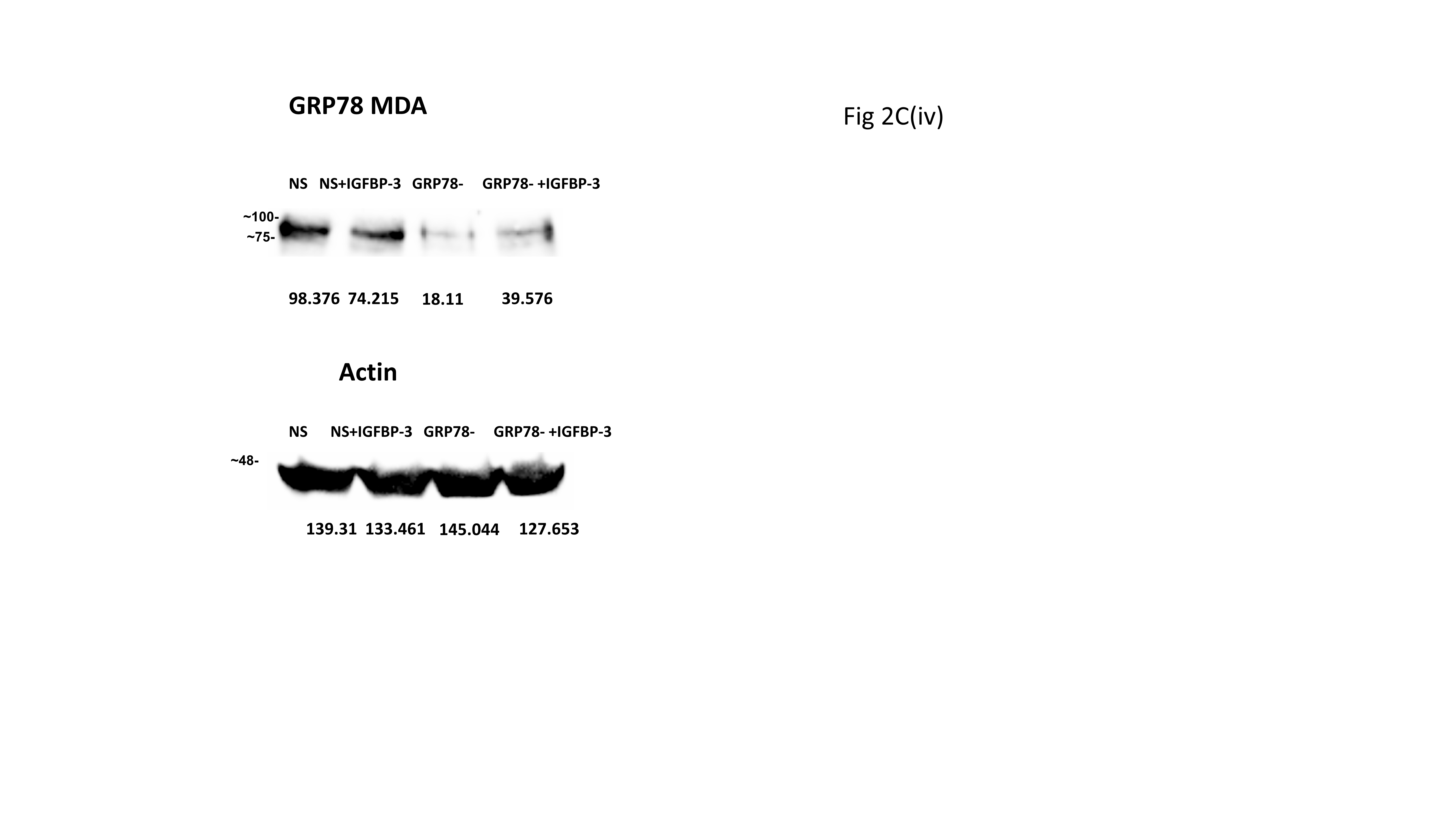

Supplement: Supplementary file 1 [file cancers-12-03821-s001.zip › original WB 20-10-30/Slide9.TIF]
